# Length-Weight Relationship, Size Distribution and Sexual Maturity of Climbing Perch, *Anabas testudineus* (Bloch, 1792) From Different Habitats in Malaysia

**DOI:** 10.21315/tlsr2026.37.1.8

**Published:** 2026-03-31

**Authors:** Mohamad Jalilah, Victor Tosin Okomoda, Khor Waiho, Hidayah Manan, Hassan Anuar, Thumronk Amornsakun and, Musa Nadirah

**Affiliations:** 1 Higher Institution Center of Excellence (HICoE), Institute of Tropical Aquaculture and Fisheries, Universiti Malaysia Terengganu, 21030 Kuala Nerus, Terengganu, Malaysia; 2 WorldFish, Ibadan, Nigeria. c/o International Institute of Tropical Agriculture (IITA), PMB 5320, Oyo Road Ibadan 200001, Oyo State, Nigeria; 3 Department of Fisheries and Aquaculture, College of Forestry and Fisheries, Joseph Sarwuan Tarka University (formerly Federal University of Agriculture Makurdi), Makurdi P.M.B. 2373 Makurdi, Nigeria; 4 STU-UMT Joint Shellfish Research Laboratory, Shantou University, Shantou, Guangdong, China; 5 Faculty of Fisheries and Food Science, Universiti Malaysia Terengganu, 21030 Kuala Nerus, Terengganu, Malaysia; 6 Centre for Chemical Biology, Universiti Sains Malaysia, 11800 Pulau Pinang, Malaysia; 7 Department of Aquaculture, Faculty of Fisheries, Kasetsart University, Bangkok, Thailand; 8 Faculty of Science and Technology, Prince of Songkhla University, Pattani campus, Amphpure Muang, 94000 Pattani, Thailand

**Keywords:** Growth, Length-Weight Relationship, Condition Factor, Reproductive Performance, Size at Maturity, Tumbesaran, Hubungan Panjang-Berat, Faktor Keadaan, Prestasi Pembiakan, Saiz pada Kematangan

## Abstract

The growth and reproductive performance of fish are influenced by ecological factors. This study investigated the biology of the climbing perch, *Anabas testudineus*, in relation to habitat. A total of 2,880 climbing perch were collected from three habitats (irrigation canal, swamp and paddy field) in Peninsular Malaysia during the dry and rainy seasons, and their biometric measurements (total length and body weight) and sexual maturity indices were measured. Two-way Classification Analysis (TCA) revealed significant sexual dimorphism in all morphometric parameters, significant habitat effects on fish size, and pronounced seasonal variation in environmental parameters, while condition factor and seasonal effects on morphometrics were not significant. Results showed that body size was largest in fish from irrigation canals during the rainy season, with males averaging 12.84 ± 1.17 cm in total length (32.38 ± 4.4 g body weight) and females averaging 13.70 ± 1.83 cm (52.84 ± 12.54 g), consistent with the upper size range reported for this species. Fish from irrigation canals also exhibited more advanced gonadal development, confirmed by higher gonadosomatic indices (GSI) and histological evidence of vitellogenic oocytes in females and spermatozoa-filled seminiferous lobules in males. Principal Component Analysis (PCA) indicated that larger body sizes and advanced gonadal development were associated with higher dissolved oxygen and pH, whereas smaller sizes correlated with elevated water temperature. This study highlights the critical roles of sex, habitat quality and seasonal variation in shaping the growth and reproductive traits of wild climbing perch populations and provides evidence-based insights for fisheries management in tropical freshwater ecosystems.

## Abstrak

Tumbesaran dan prestasi pembiakan ikan dipengaruhi oleh faktor-faktor ekologi. Kajian ini menyelidik beberapa aspek biologi ikan puyu, Anabas testudineus yang berkaitan dengan habitat mereka. Sejumlah 2,880 ikan puyu telah dikumpulkan dari tiga habitat (iaitu terusan pengairan, paya dan sawah padi) di Semenanjung Malaysia semasa musim panas dan hujan, dan parameter morfometrik serta indeks kematangan seksual mereka diukur dan dianggarkan. Keputusan menunjukkan perbezaan yang signifikan (P < 0.05) dalam kebanyakan parameter antara ikan jantan dan betina, serta merentasi habitat dan musim. Saiz badan ikan puyu yang disampel semasa musim hujan dari terusan pengairan adalah paling besar berbanding musim panas dan habitat lain (jantan, 12.84 ± 1.17 cm panjang keseluruhan dan 32.38 ± 4.4 g berat badan; betina, 13.70 ± 1.83 cm panjang keseluruhan dan 52.84 ± 12.54 g berat badan). Ikan dari terusan pengairan semasa musim hujan menunjukkan saiz badan terbesar dan perkembangan gonad yang lebih bagus, seperti yang dibuktikan oleh indeks gonadosomatik (GSI) yang lebih tinggi dan gamet yang terbeza dengan baik berbanding dengan ikan dari habitat lain. Trend kualiti air menunjukkan bahawa saiz badan yang lebih besar dikaitkan dengan tahap oksigen terlarut (DO) dan pH yang lebih tinggi, manakala saiz badan yang lebih kecil dikaitkan dengan suhu air yang tinggi. Kajian ini menekankan peranan penting kualiti habitat, variasi bermusim, dan jantina dalam membentuk ciri-ciri tumbesaran dan pembiakan populasi ikan puyu liar.

**HIGHLIGHTS**

- Growth and reproductive performance of climbing perch were influenced by habitat type and seasonal variations.
- Fish in irrigation canals during the rainy season exhibited the largest size and enhanced gonadal development.
- High dissolved oxygen and pH were correlated with larger fish, while rising temperatures were linked to smaller sizes.
- Findings underscore the importance of habitat quality and seasonal changes in shaping wild climbing perch populations.

## INTRODUCTION

The climbing perch, *Anabas testudineus* (Bloch, 1792) (Perciformes: Anabantoidei: Anabantidae) is a commercially important fish in Malaysia, with a market price ranging between RM18 and RM20 per kilogram (Chaturvedi *et al*. 2015; Marimuthu *et al*. 2009a; Hassan 2017; Syazwani 2020) and highly nutritious (Uttam *et al*. 2005; Tay *et al*. 2006; Mohanty *et al*. 2016; Paul *et al*. 2017; Akter *et al*. 2020). Besides being a protein source, the species also serves as a biological control agent for mosquito larvae, including Aedes, Anopheles and Culex, due to its tolerance of poor water quality (Hussain 2005; Chandra *et al*. 2008; Khairul 2011).

Climbing perch are found mostly in freshwater ecosystems; inhabiting habitats such as paddy fields, swamps, reservoirs, ditches, ponds, marshland, canals and irrigation canals, as well as streams that are filled with vegetation (Iwata & Kiguchi 2003; Das *et al*. 2009; Froese & Pauly 2019). They possess labyrinth organs enabling aerial respiration and can withstand drought by burrowing into mud (Jacob 2005; Hitchcock 2008; Morioka *et al*. 2009b). However, wild populations are declining due to habitat destruction caused by overfishing, urbanisation, agriculture, industry and pollution (Khatune-Jannat *et al*. 2012; Hossain *et al*. 2015).

Knowledge of their growth and reproduction is vital for developing breeding techniques and improving seed production (Kagawa 2013). Many studies have examined reproductive biology, including gonadal development (Uddin *et al*. 2016; Priyatha & Chitra 2022), fecundity (Marimuthu *et al*. 2009b), breeding behaviour (Zworykin 2012), larval morphology and growth (Amornsakun *et al*. 2005; Morioka *et al*. 2009a), culture systems (Rahman & Marimuthu 2010; Uddin *et al*. 2016; Anantharaja *et al*. 2017), feeding requirements (Hossain *et al*. 2012) and disease control (Sumon *et al*. 2015; Tran *et al*. 2024; Shahriar *et al*. 2024). Yet, there remains limited information on ecological interactions such as water quality, habitat diversity and climate influences.

The capability of a species to reproduce successfully under a rapidly changing environmental condition is crucial to ensuring its continuity in an ecosystem (Moyle & Crech 2000; Le Doux-Bloom *et al*. 2021; Morales-Gamba *et al*. 2021). This is because the life history strategies such as development, growth and reproduction depend largely on the environmental conditions of the habitat (Magalhaes *et al*. 2003; Olden *et al*. 2006). Distinct ecological variables drive adaptive strategies involving developmental, behavioural and physiological changes (Andrade & Braga 2005; Amtyaz Khan *et al*. 2013; Fafioye & Ayodele 2018; Ahmadi 2019).

A study of the length-weight relationship (LWR), size distribution and sexual maturity of climbing perch is essential to assess the growth patterns, reproductive potential and overall health of the population on this species, which plays a vital role in sustainable management as well as aquaculture practices. According to Ndobe *et al*. (2019), LWR reveals growth patterns, whether isometric (weight proportional to length) or allometric (weight grows disproportionately). This also relates to condition factor, an indicator of population health (Khatune-Jannat *et al*. 2012). LWR knowledge supports aquaculture by identifying optimal conditions for growth such as diet and environment (Hamid *et al*. 2015). Size distribution provides insight into age structure and recruitment success, while skewed distribution may reflect overfishing or habitat stress (Wilson *et al*. 2010).

Moreover, the investigation of sexual maturity is basic to estimating the reproductive capacity of the species. Because life history information such as the age and size at first maturity speaks to population resiliency, it is critical for establishing successful harvesting regulations including the minimum size limit set to avoid the take of immature individuals, thus enabling the population to be replenished (Cerdenares-Ladrón *et al*. 2011; Perez-Palafox *et al*. 2022). In aquaculture, the utilisation of climbing perch reproductive biology information facilitates control of breeding programs for maximum production and sustainability (Mawa *et al*. 2022). Sexual maturity also reflects environmental effects such as temperature (Kuparinen *et al*. 2011), water quality (Mariu *et al*. 2023) and food availability (Yoneda & Wright 2005), supporting both aquaculture and conservation.

Similarly, tracking LWR, size distribution and sexual maturity can provide insight for developing targeted management plans, such as seasonal fishing bans or the designation of protected breeding grounds (Russell *et al*. 2011). Due to the wide distribution range of climbing perch throughout Southeast Asia and its importance in both subsistence and commercial fisheries (Kumar *et al*. 2012), this is particularly relevant. Knowing these biological parameters in aquaculture is essential for effective stock management, improving breeding efficiency and making production practices sustainable and environmentally friendly (Rahman & Samat 2021). Thus, these parameters are indispensable for improving fishery sustainability and conservation of climbing perch.

Other factors such as food availability/quality, population density, predation force, physical and physiological stressors as well as disease threats also play important role in shaping a species’ physiological characteristics (Khatune-Jannat *et al*. 2012; Abdel-Tawwab *et al*. 2015; Ndobe *et al*. 2019). Yet, information on climbing perch remains limited, especially in Malaysia, despite extensive studies in other Asian countries (Mukherjee *et al*. 2003; Jacob 2005; Sarkar *et al*. 2005; Atal *et al*. 2009; Morioka *et al*. 2009b; Kumar *et al*. 2013; Ahmadi 2019). Therefore, this study was designed to investigate the size distribution, LWR, and maturity of climbing perch across different seasons and habitats of Peninsular Malaysia. Findings will provide baseline data on growth, maturity and spawning, serving as reference for stock assessment, management and conservation.

## MATERIALS AND METHODS

### Sampling Site and Condition

**FIGURE 1:**
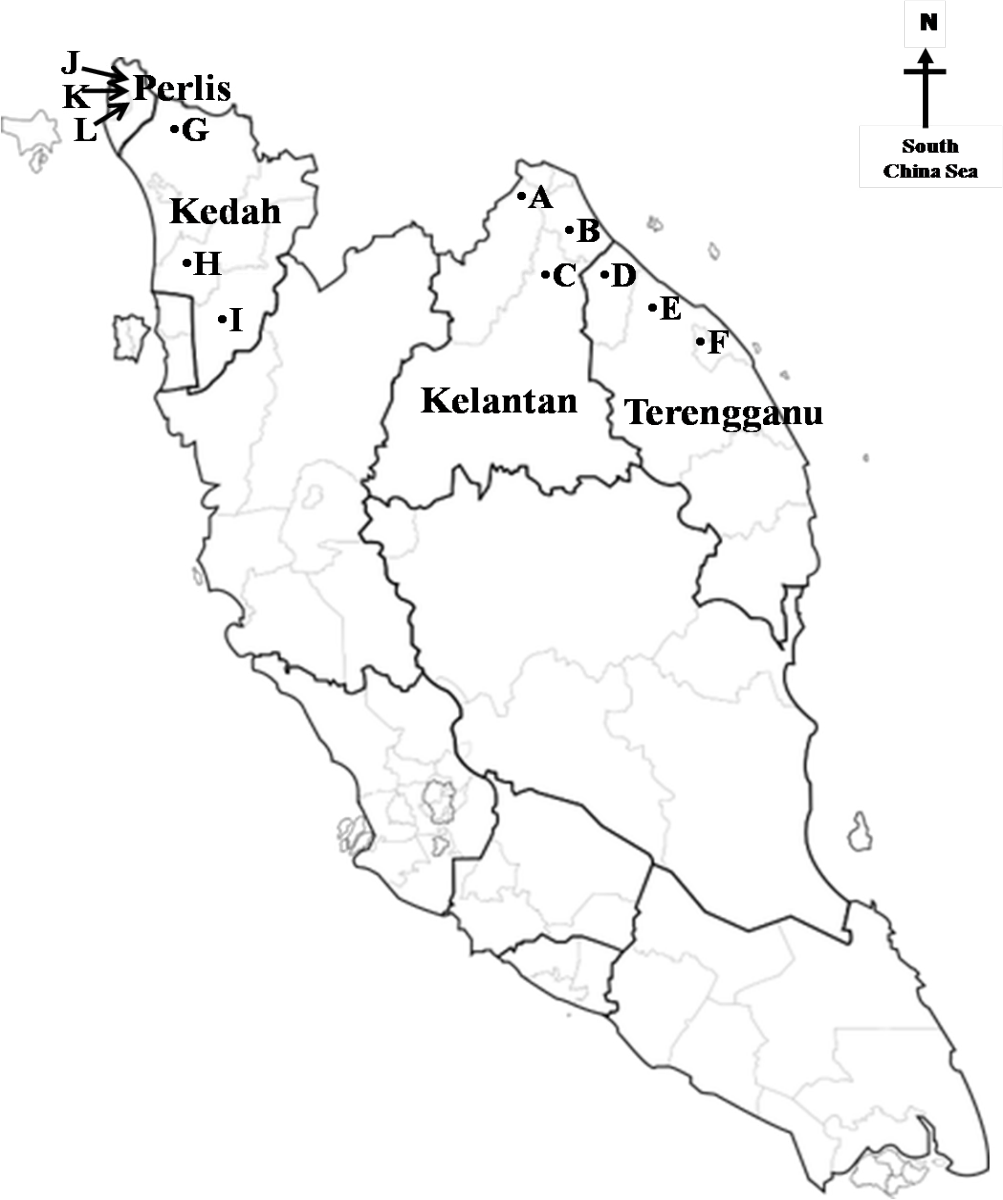
Map of Peninsular Malaysia showing the sampling locations across the eastern and northern states. In Kelantan, the sites were (A) Tumpat, (B) Bachok and (C) Pasir Putih, while in Terengganu the sites included (D) Besut, (E) Setiu and (F) Kuala Terengganu. In the northern region, sampling was conducted in Kedah at (G) Kubang Pasu, (H) Kuala Muda and (I) Kulim, and in Perlis at (J) Beseri, (K) Padang Besar and (L) Mata Ayer.

A total of 2,880 adult-sized climbing perch (1:1 male-to-female ratio) were collected from four states in Malaysia. Two states from the north-eastern [i.e., Kelantan (5.2500°N, 102.0000°E) and Terengganu (4.7500°N, 103.0000°E)], and north-western Peninsular Malaysia [i.e., Kedah (6.1283° N, 100.3628°E) and Perlis (6.5000°N, 100.3217°E)] were selected for this study (Fig. 1). A total of 60 fish per state were captured by trapping (Trap size; 2 × 2 × 2 feet and mesh size of 1 inch) angling (hook size; 10–12 and worm as bait) and netting (mesh size; 1 inch) monthly over a period of one-year (both dry and rainy season). According to the Malaysian Meteorological Department (2012; 2013), the dry season in the eastern and northern regions of Peninsular Malaysia was observed from February to July and from January to June, respectively. The rainy season, on the other hand, extended from August to January in the eastern region and from July to December in the northern region. The physico-chemical characteristic of water (water temperature, dissolved oxygen and pH) in each sampling site were recorded *in-situ* using an hand-held YSI meter (YSI model 556 MPS, USA) in three replicates.

### Sampling Habitats

At the different states, fish were captured from three habitats namely, paddy fields, irrigation canals and swamps. Paddy field selected are paddy cultivation areas with a water depth of about 0.40 ± 0.05 m with soft, muddy substrate (following habitat descriptions adapted from Das 2002). The irrigation canals selected were 1.65 ± 0.09 m in water depth. They have muddy sandy substrate while the sides of the canals are dominated with water grasses (similar to the methods described by Katano *et al*. (2003) and Thompson and Larsen (2004). The swamp area selected have a static cloudy water with depth of about 1.70 ± 0.12 m. The substrates are mostly muddy, covered by woody debris (based on standard sampling habitat classification in Boyd and Tucker [1992] and Christensen *et al*. [1996]). The water depths reported reflect the natural and typical conditions of each habitat during the sampling period, ensuring ecological relevance to the species’ natural environment.

### Sex Identification and Biometric Characteristic Measurement

**FIGURE 2:**
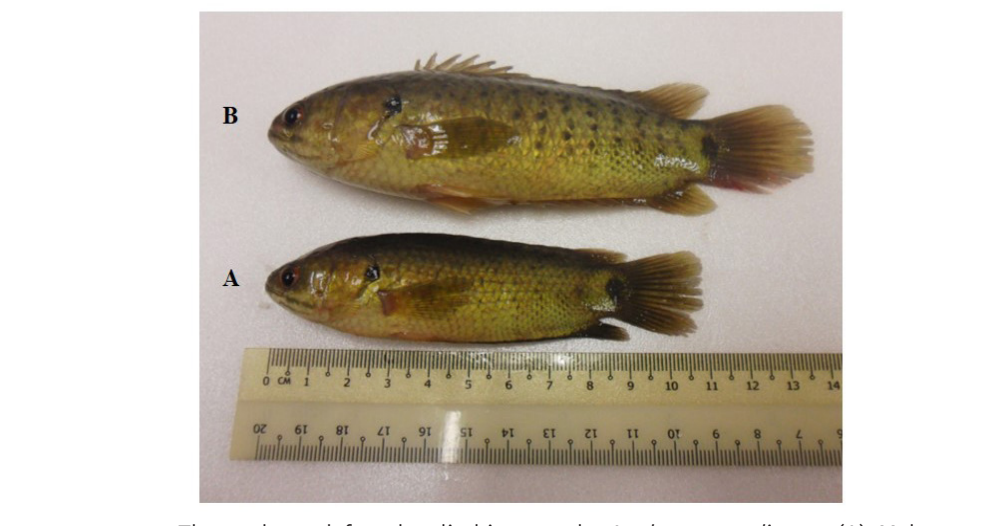
The male and female climbing perch, *Anabas testudineus*. (A) Male; the male’s body was thinner with flat abdomen and smaller in size, (B) Female; the female was bigger in size, while abdomen was bulgy and soft in appearance especially during the matured stage of ovaries.

After sampling, the sex of the fish was identified *in-situ* primarily based on the morphology of the genital papilla (Fig. 2) (Nyakuni 2005; Ernawati *et al*. 2009; Mawa *et al*. 2022). In females, the genital papilla appeared slightly pink and swollen, and in some cases, eggs were released when gentle pressure was applied to the abdomen. In males, the genital papilla was pointed and narrow, with free oozing milt upon slight abdominal pressure. Although body shape and abdominal features (e.g., swollen abdomen in females or elongated body in males) were observed, these were considered supportive traits and not the primary criteria for sex identification, particularly since immature females are difficult to distinguish from males based on external body features alone. In cases where sex determination was uncertain, representative samples were further validated through gonadal histological examination to ensure accuracy in sex identification and subsequent biological indices (e.g., GSI and HSI).

Individual body weight was measured using OHAUS OH-400 digital balance to 0.01 g while the lengths were measured using a stationery meter ruler to 0.01 cm. The total length of the fish was measured from the tip of the snout to the tip of the longer lobe of the caudal fin (Hossain *et al*. 2017). Thereafter, the fish abdomen was dissected, and the gonads and liver were carefully removed and weighed using a digital balance. The histological analysis of the gonads is presented in Fig. 5. The gonad was weight and fixed in 10% buffered formaldehyde at 1:10 (sample to fixative) for histological analysis. The bottles containing specimen were sealed tightly and labelled. The liver was also weighed and sealed in a small plastic bag and stored in an ice box for transportation to the university campus where histological analysis was made. All the procedures were carefully carried out based on the methods of Layton *et al*. (2018), Furnesvik *et al*. (2022) and Tian *et al*. (2024).

### Histological Assay

The gonads (testes and ovaries) were subjected to histological analysis following the procedure adopted by Suchiang and Gupta (2011). The histological procedures included the specimen preparation, fixation, tissue processing, embedding, cutting, and hematoxylin and eosin (H&E) staining. The histological results were viewed under microscope and all the microscopic images were captured using a Dino-capture software. The oocytes were classified according to Lubzens *et al*. (2010) into previtellogenic, vitellogenic and maturation phase, whereas the sperms were categorised according to Cek and Yilmaz (2007) and Schulz *et al*. (2010) as spermatogonia, spermatocytes, secondary spermatocytes, spermatid and spermatozoa.

### Calculation and Statistical Analysis

Statistical analyses for the length-weight relationship of climbing perch were performed using Microsoft Excel 2010 and was calculated by using the expression adopted by Ondhoro *et al*. (2017) and Fafioye and Ayodele (2018):


$$\text{W}={\text{aL}}^{\text{b}}$$

where W = body weight (g); L = total length (cm); a = intercept and b = regression coefficients.

The BW and TL of the individual fish were log-transformed and subjected to linear regression analysis (log BW = log a + b log TL).

Fulton’s condition factor ‘K’ for the fish was calculated using the formula adopted from De-Giosa *et al*. (2014):


$$K=\frac{W}{L^{3}}\times 100$$

where *K* = condition factor, *W* = body weight of fish and *L*^3^ = body length.

The body sized and water quality parameters data were subjected to a multivariate analysis of variance (MANOVA), at a significance level of α = 5%, followed by Bonferroni correction for post hoc comparisons. Student’s *t*-test were also applied to determine the size comparison between season and sex, while one-way ANOVA are conducted to define the size comparison among habitat. A post-hoc Tukey’s test was applied if significant differences between subjects occurred and results were considered as statistically significant at the *p* < 0.05 level. In addition, Pearson’s correlation test was used to explore the relationship between parameters. Furthermore, a Two-Component Analysis (TCA) was performed to visualise the relationship between fish morphology and water quality parameters.

## RESULTS

The water quality parameters range for temperature, dissolved oxygen and pH were 26.38 ± 0.14 – 32.50 ± 0.26°C; 3.57 ± 0.79 – 7.26 ± 1.54 mg/L; and 5.15 ± 0.50 – 6.96 ± 0.28, respectively. The TL of male climbing perch used in the present study ranged from 10.66 cm to 12.23 cm and the BW ranging from 23.12 g to 29.28 g. Whereas the female TL ranged from 11.11 cm to 12.77 cm, while the BW ranging from 31.61 g to 46.57 g (Table 1). The fish sampled from the paddy field are the smallest size in males and female for the TL and BW in this study. Results of student’s *t*-test analysis of size between season and size between sexes was significantly difference (*p* < 0.05). Similarly, comparison between different habitats also was indicative that the size of climbing perch was significantly different (*p* < 0.05). Although the differences in TL and BW between habitats were relatively small, fish sampled from the paddy field were significantly smaller than those from irrigation canals and swamps, as supported by statistical analysis (*p* < 0.05).

The mean TL and BW of climbing perch was significantly smaller in males than females in all habitats (*p* < 0.05). BW of this fish was significantly heavier during rainy season compared to dry season (*p* < 0.05), but the TL was not significantly difference (*p* > 0.05) (Table 1). On a general note, the size of fish obtained during the rainy season was larger compared with those obtained during the dry season. The condition factor revealed that female was significantly (*P* < 0.05) larger than male regardless of the season and habitats (Table 1). Higher condition factor was recorded in the male fish harvested from paddy field at the dry (1.91) and rainy season (1.80). While in the females, the highest condition factor was noticed in swamps habitat during the dry (2.57) and rainy season (2.72).

**FIGURE 3:**
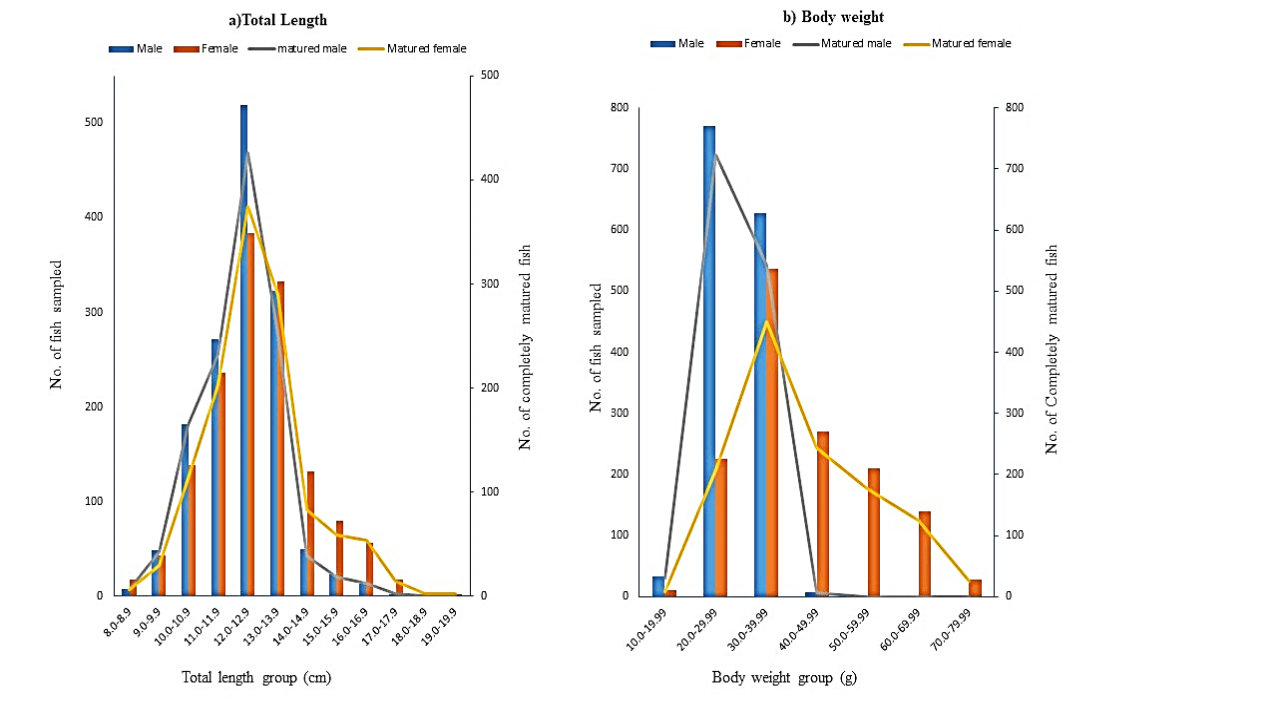
Size distribution of male and female climbing perch, *Anabas testudineus* based on their (a) total length and (b) body weight as well as gonadal maturity.

The size distribution of the climbing perch sampled for this study is as shown in Fig. 3. Majority of the fish were within the length and weight range of 12.0 cm to 12.9 cm and 20.0 g to 29.99 g, respectively. The lowest number of the fish were accounted within the extremes of the sizes observed in this study. The size of matured male climbing perch ranged between 12.0 cm–12.9 cm TL and 20.0 g–29.99 g BW, while females ranged from 12.0 cm–12.9 cm TL and 30.0 g–39.99 g BW (Fig. 3). The frequency of matured climbing perch in different habitats (Tables 2 and 3) refers to the first maturity stage (M₁), defined as the smallest size at which gonads are functionally mature, with males exhibiting developed testes containing spermatozoa and females showing vitellogenic oocytes. Result obtained showed smaller size (i.e., length and weight) of matured fish were caught in the Paddy field while the irrigated canal had the largest number of large fishes. In all habitats, the mature female samples were more than the males.

**FIGURE 4:**
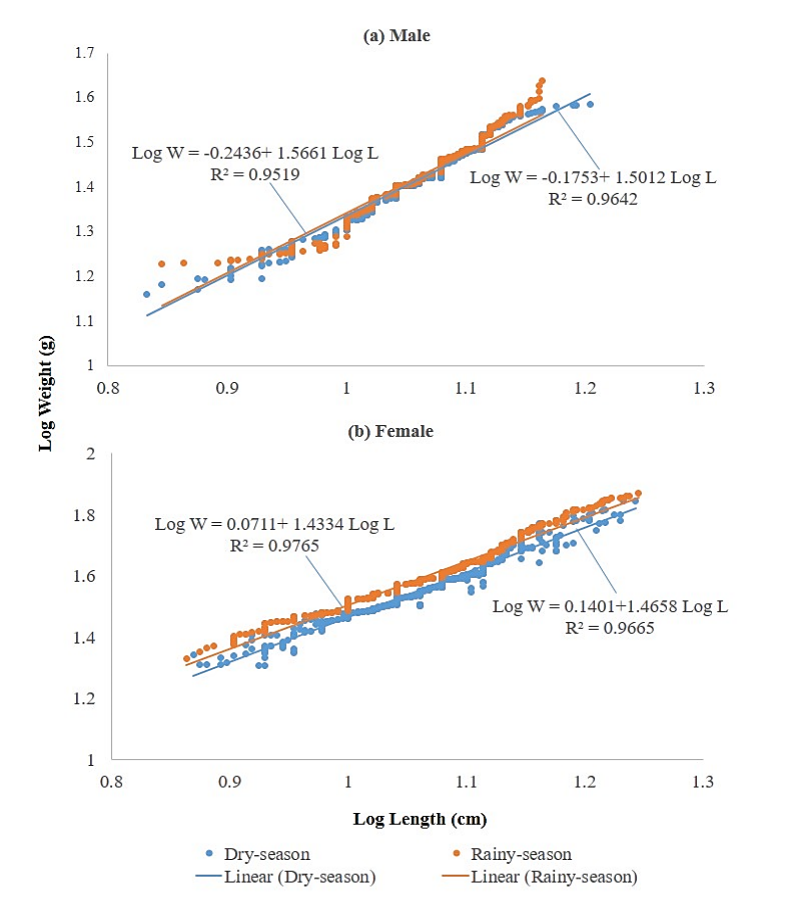
Length-weight relationship of (a) male and (b) female climbing perch, *Anabas testudineus* in Malaysian waters.

The log transformed length-weight relationship is as shown in Fig. 4 and Table 4. The growth pattern of the climbing perch was determined based on the total length–body weight relationship. Negative allometric growth (b < 3) was observed for both sexes during dry and rainy seasons, indicating that body weight increased at a slower rate than total length. No other morphometric traits were included in this analysis. The Intercept (a) and regression value (r^2^) of the length-weight relationship graph ranges from −2.244 to 1.140 and 0.952 to 0.952, respectively.

The growth pattern of the climbing perch was determined based on the total length–body weight relationship. Negative allometric growth (b < 3) was observed for both sexes during dry and rainy seasons, indicating that body weight increased at a slower rate than total length. No other morphometric traits were included in this analysis.

The Pearson correlation coefficient, *r* (Table 5), indicated that water temperature had a strong negative correlation with the body size of the climbing perch (*p* < 0.05, *r* > −0.8), while dissolved oxygen and pH showed moderate positive correlations (*p* < 0.05, *r* = 0.518–0.652). These results suggest that higher water temperatures were associated with smaller body sizes, whereas higher DO and pH were associated with larger body sizes. It is important to note that these correlations reflect statistical associations rather than direct causal relationships, and do not imply that simultaneous increases in all water quality parameters would automatically lead to increased body size. Other environmental and biological factors, such as habitat type, food availability and intraspecific competition, may also influence growth.

The TCA analysis revealed significant sexual dimorphism across all morphometric parameters (Table 6). Female fish consistently exhibited greater total length, higher body weight, and elevated condition factor compared to males (*p* < 0.01), with body weight showing the largest difference, averaging 46% heavier than males. No significant seasonal effects were detected on any morphometric parameters (*p* > 0.05), indicating stable growth across dry and rainy seasons. Habitat type significantly influenced fish size, with individuals from irrigation canals exhibiting the largest total length and body weight, followed by swamps and paddy fields. Post-hoc analysis confirmed significant pairwise differences among habitats (*p* < 0.05). Condition factor did not vary significantly among habitats (*p* > 0.05). Environmental parameters showed pronounced seasonal variations, with dry season temperatures significantly higher than those in the rainy season, while dissolved oxygen levels were elevated during the rainy season (*p* < 0.01). Dissolved oxygen also differed among site types, with ditches showing higher levels compared to ponds and paddy fields. No significant interaction effects were observed for any parameter.

**FIGURE 5:**
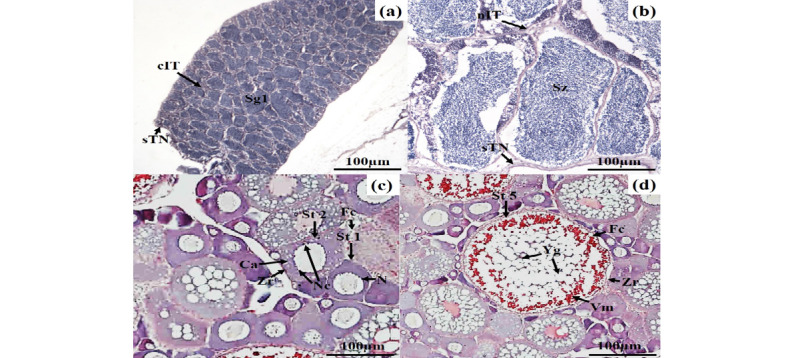
The spermatogenesis in the testis of male climbing perch, *Anabas testudineus*. (a) The sperm cells in the spermatogonia phase (Immature); (b) The sperm cells in spermatozoa phase (Matured); and the oocyte stage in the ovary of female climbing perch, *A. testudineus*; (c) Oocyte of stage 1 (St1) and stage 2 (St2) in the previtellogenic phase (Immature); (d) Oocyte of stages 5 (St5) in the maturation phase (matured). Labelled features include: Sg1 = spermatogonia, cIT = continuous interstitial tissue, sTN = Simple tunica, Sz = spermatozoa, nIT = non-continuous interstitial tissue, N = nucleus, Nc = nucleolus, Ca = cortical alveoli, Fc = follicular cell, Zr = zona radiate, Yg = yolk globules, Vm = vitelline membrane. Scale bar = 100 μm.

The histological analysis of gonads (Fig. 5) allowed clear classification of male spermatogenesis into two stages (immature and mature) and female oogenesis into five stages (St1–St5). Based on this classification, body weight data were sorted according to reproductive stage for each habitat and season (Table 3). In males, fish weighing 10 g–30 g were predominantly in the immature or developing stage, while those above 30 g were mostly mature, a pattern consistent across all habitats. In females, lower body weight groups (10 g–20 g) corresponded to pre-vitellogenic oocytes (St1–St2), whereas higher body weight groups (30 g–60 g) were associated with vitellogenic and mature oocytes (St4–St5). Notably, the highest female body weights were observed in swamp and irrigation canal habitats during the rainy season. These results demonstrate a clear linkage between body weight, reproductive stage, habitat and seasonal variation, indicating that gonadal maturation is influenced by both environmental conditions and individual growth. The revised Table 3 now explicitly presents reproductive stages alongside body weight to support these conclusions.

## DISCUSSION

Both biotic and abiotic factors affect the physiological index of fish in the wild or culture under captivity (Iwata & Kiguchi 2003 ). This study has demonstrated the influence of abiotic factors such as habitats and seasons on the size distribution of climbing perch in the wild. The differences in these abiotic factors can influence water quality (such as temperature, dissolved oxygen and pH), as well as fish shelter, food availability, predation pressure and susceptibility to disease (Craig 2015; Gebrekiros 2016; Le Doux-Bloom *et al*. 2021). In this study, we focused on temperature, dissolved oxygen and pH, which are widely recognised as the primary water quality parameters affecting fish growth and reproduction in freshwater systems (Boyd 2015; Kumar *et al*. 2012), while other parameters such as hardness and alkalinity were not measured but may also have ecological relevance. The findings of this study suggest that larger climbing perch (defined here as individuals in the upper quartile of total length and body weight) were mainly found in the rainy season, possibly due to higher precipitation which improved the water quality in the habitat (dissolved oxygen range of 4.0 mg/L to 7.3 mg/L; pH range of 5.3 to 6.9). These values fall within the recommended ranges for optimal growth and reproduction of the species (Boyd 2015; Chakraborty & Haque 2014). Higher dissolved oxygen levels can enhance metabolic activity and feeding efficiency, while pH within the optimal range reduces physiological stress, both of which can contribute to larger body size. Additionally, warmer water temperatures during the rainy season may increase metabolic rate and growth, although direct evidence in the climbing perch is limited. In the same light, previous studies (Chifamba 2000; Patrick 2016) highlighted that increases in water level and improved water quality during the rainy season are associated with the presence of larger-sized freshwater fish in natural habitats.

The mean body size of climbing perch was significantly smaller in males than females in all habitats examined, based on mean total length and body weight, with statistical significance confirmed using independent *t*-tests (*p* < 0.05). Females were consistently larger and heavier, particularly during the breeding season, which aligns with observations in previous studies reporting specific size ranges for male and female climbing perch (Zalina *et al*. 2012; Begum & Minar 2012; Ndobe *et al*. 2019). To further support this, additional morphometric measurements such as body depth, head length, and eye diameter were log-transformed to assess allometric growth and sexual dimorphism. The tendency for females to attain larger sizes is common in climbing perch species from other regions and is often linked to reproductive investment and energy allocation. In fact, Pauly (2019), emphasised the fact that about 80% of matured female sampled are bigger than the males. At spawning season, the heavier body weight of the female fish can be easily explained by the increased gonad weight with fully developed oocytes (Jacob 2005; Amornsakun *et al*. 2005). Growth is said to be positive allometric when the weight of an individual species increases than the length (b > 3) and negative allometric when length increases over the weight (b < 3) (Frose 2006; Kumar *et al*. 2013). In the present study, the slope (b) of the length–weight relationship, calculated from our own data, was highest in males during the rainy season and lowest during the dry season, whereas in females, the opposite trend was observed. All slope values across habitats and seasons were significantly lower than the isometric value of 3 (*p* < 0.05), indicating negative allometric growth; hence, climbing perch became slenderer as total length increased. This finding is consistent with previous observations in freshwater species (Rathod *et al*. 2011), but the values reported here are specific to the populations and conditions studied. This is similar to the reported of many previous studies by Kumar *et al*. (2013), Hossain *et al*. (2015), Kumary and Raj (2016), Ahmadi *et al*. (2018) and Ahmadi (2019).

In contrast, wild population of climbing perch in Indonesia (Ernawati *et al*. 2009; Ndobe *et al*. 2019), as well as captive cultured of this species in Bangladesh (Begum & Minar 2012) have been reported to exhibit an isometric growth pattern. The growth pattern of a fish species can be influenced by many factors ranging from environmental, to nutritional status (Izquierdo *et al*. 2001), sex reproductive activity (Petersen & Warner 2002; Pankhurst & Porter 2003), inherited genetics, population density, predation force, physical and physiological stresses and disease threats (Magalhaes *et al*. 2003; Olden *et al*. 2006; Pankhurst *et al*. 2008). According to Barnham and Baxter (1998), if the K value is 1.00, the fish condition is poor, long and thin; if the K value is 1.20, the fish condition is moderate; if the K value 1.40, the fish condition is good and well-proportioned; and if the K value 1.60, the fish condition is excellent. Interestingly, the value of K in climbing perch in different seasons and by sexes in this study was over the value of 1.60 which indicated that, they were in outstanding condition. This is therefore indicative of good state of health, excellent growth rate and suitable feeding intensity, despite the fish having a negative allometric growth pattern as stated earlier. The higher condition factor (K) observed in female samples, particularly during the rainy season, is associated with advanced oocyte stages (St4–St5) as indicated by histological analysis, reflecting increased gonad weight during vitellogenesis and maturation. This finding supports the linkage between reproductive stage, body weight and seasonal variation in habitat conditions. Morato *et al*. (2001) and Gupta *et al*. (2011) stated that the food availability at a particular time, the type of food consumed, the fullness of gut, amount of fat reserve and amount of muscular development also led to the differentiation of the K value.

The observed sexual dimorphism, with females larger and heavier than males, aligns with previous studies where females allocate more energy toward reproduction (Im *et al*. 2016; Mollet *et al*. 2023). The absence of seasonal effects on morphometrics suggests behavioural and physiological adaptations allowing consistent growth (Dubuc *et al*. 2024). Habitat influenced total length and body weight, likely due to differences in water flow (Li *et al*. 2023), nutrient availability (Stasko *et al*. 2016; Liu *et al*. 2024), and competition, while the consistent condition factor indicates proportional growth and ecological adaptability (Li *et al*. 2023). Seasonal and habitat-related variations in temperature and dissolved oxygen reflect patterns in tropical freshwater systems, highlighting the importance of monitoring environmental parameters for fisheries management (Bowden *et al*. 2014; Kominoski *et al*. 2021). The lack of significant interaction effects suggests that sex, season and habitat act independently, enabling straightforward management strategies such as habitat conservation and sex-specific population assessments (Piferrer & Guiguen 2008; Nakagawa 2019).

The results showed that male and female climbing perch reached first sexual maturity at a length class of 12.0 cm–12.9 cm, corresponding to a weight class of 20.00 g–29.99 g for males and 30.00 g–39.99 g for females, as confirmed by histological analysis of gonads. These values are consistent with previous reports of first maturity sizes in climbing perch from other freshwater systems (Zalina *et al*. 2012; Begum & Minar 2012), supporting their use as indicators of sexual maturity in the populations studied. This is in contrast with Jacob (2005), who reported that this species from India matured at 9.1 cm TL; however, our sample did not include many smaller individuals, and the observed first maturity sizes of 12.0 cm–12.9 cm TL reflect the size class at which most individuals in our population reached gonadal maturity, as confirmed by histological analysis. Future studies with broader sampling including smaller juveniles could provide a more precise estimate of first maturity size. The majority (> 95%) of testes and ovary in male and female climbing perch have reached matured phase during the rainy season while mostly immature phase or resting stage were observed during the dry season. Thus, our findings suggest that the majority of climbing perch spawn from November to February in the eastern states and September to December in the northern states of Peninsular Malaysia, with variation across different habitats such as irrigation canals, swamps and paddy fields, likely influenced by local water availability and monsoon patterns. This period generally coincides with the end of the rainy season and beginning of the dry season, although some natural habitats may occasionally dry out completely during intense droughts (Schuler 2014; Zalina *et al*. 2012; Patrick 2016). Even though climbing perch can burrow into the muddy sediment and hibernate during the drought season (Hitchcock 2008), they are not able to move or eat during that period, and their reproductive organs are in resting stage because of the scarce energy needed to mostly maintain life, hence their development and growth is halted (Ultsch 1989; Kjesbu *et al*. 1998). This therefore explains the reduce reproductive functionality in the dry season compared to the raining season.

## CONCLUSION

This study provides comprehensive insights into the growth and reproductive biology of climbing perch across different habitats and seasons in Peninsular Malaysia. The findings indicate that males and females exhibit negative allometric growth, with females generally attaining larger body size and higher condition factor (K), particularly during the rainy season. Reproductive stages were clearly linked to body weight, seasonal variation and habitat type, with males maturing predominantly at 12.0 cm–12.9 cm TL and 20.00 g–29.99 g, and females at 12.0 cm–12.9 cm TL and 30.00 g–39.99 g, corresponding to first sexual maturity. Spawning periods varied between regions, with habitat-specific differences influenced by local water availability and monsoon patterns, generally coinciding with the end of the rainy season and start of the dry season. These results highlight the importance of considering both environmental factors and individual growth in fisheries management, and provide baseline data for future studies on population dynamics, habitat conservation and aquaculture of climbing perch in Malaysia.

## Figures and Tables

**TABLE 1:** Mean total length (TL), body weight (BW) and condition factor (K) of male and female climbing perch, anab ccc

Measurement	Sex	Season	Habitat		

			Irrigation canal	Swamp	Paddy field
Total length (cm)	Male	Dry	12.01 ± 1.03^a^	11.53 ± 1.05^b^	10.66 ± 1.31^c^
		Rainy	12.23 ± 0.99^a^	11.63 ± 0.64^b^	11.02 ± 1.26^c^
	Female	Dry	12.75 ± 1.37^a^	11.43 ± 1.50^b^	11.11 ± 1.56^c^
		Rainy	12.77 ± 1.51^a^	11.64 ± 1.35^b^	11.34 ± 1.66^c^
Body weight (g)	Male	Dry	29.28 ± 3.56^a^	26.06 ± 3.49^b^	23.12 ± 2.80^c^
		Rainy	29.27 ± 3.51^a^	27.13 ± 3.22^b^	24.06 ± 3.37^c^
	Female	Dry	42.89 ± 5.37^a^	38.4 ± 4.66^b^	31.61 ± 3.01^c^
		Rainy	46.57 ± 5.80^a^	42.96 ± 4.00^b^	34.25 ± 2.86^c^
Condition factor	Male	Dry	1.69 ± 0.33^a^	1.70 ±0.13^b^	1.91 ± 0.21^c^
		Rainy	1.60 ± 0.18^a^	1.73 ± 0.44^b^	1.80 ± 0.25^c^
	Female	Dry	2.07 ± 0.37^a^	2.57 ± 0.49^b^	2.31 ± 0.37^c^
		Rainy	2.24 ± 0.28^a^	2.72 ± 0.19^b^	2.35 ± 0.15^c^

*Note:* Data are expressed as mean ± SD. Means values indicated by ‘b’ and ‘c’ are significantly different from the values shown by ‘a’ (ANOVA; (*P* < 0.05). Values that do not share the same letter are significantly different.

**TABLE 2:** Frequency of total length at maturity of climbing perch, *Anabas testudineus* in different habitats (*n* = 2,880).

Total length (cm)	Paddy field		Irrigation canal		Swamp	

	Male	Female	Male	Female	Male	Female
8.0–8.9	4	4	-	1	-	3
9.0–9.9	25	15	-	1	-	6
10.0–10.9	56	47	12	5	22	23
11.0–11.9	57	59	24	20	60	39
12.0–12.9	64	63	68	55	102	69
13.0–13.9	18	39	70	67	63	62
14.0–14.9	4	8	17	40	4	18
15.0–15.9	-	5	10	35	1	8
16.0–16.9	-	6	7	29	-	-
17.0–17.9	-	-	1	7	-	-
18.0–18.9	-	-	-	1	-	-
1.09–19.9	-	-	-	1	-	-

**TABLE 3:** Frequency of body weight at maturity of climbing perch, *Anabas testudineus* in different habitats (*n* = 2,880).

Body weight (g)	Paddy field		Irrigation canal		Swamp	

	Male	Female	Male	Female	Male	Female
10.00–10.99	9	-	3	-	5	5
20.00–20.99	180	12	62	1	134	85
30.00–30.99	39	164	157	56	118	113
40.00–40.99	-	76	4	80	-	51
50.00–50.99	-	-	-	48	-	53
60.00–60.99	-	-	-	53	-	9
70.00–70.99	-	-	-	18	-	1

**TABLE 4:** Length-weight relationship of male and female climbing perch, *Anabas tetsudineus* in dry and rainy season.

Parameter	Male		Female	

	Dry season	Rainy season	Dry season	Rainy season
a	−0.175	−2.244	0.140	0.071
b	1.501	1.566	1.466	1.433
r^2^	0.964	0.952	0.967	0.977

**TABLE 5:** Pearson correlation coefficient of morphological parameters of *Anabas testudineus* in relation to water quality parameter.

Sex	Male		Female	

Body parameter	Total length	Body weight	Total length	Body weight
Water quality				
Water temperature	−0.883 (*p* < 0.05)	−0.891 (*p* < 0.05)	−0.875 (*p* < 0.05)	−0.889 (*p* < 0.05)
pH	0.548 (*p* < 0.05)	0.607 (*p* < 0.05)	0.597 (*p* < 0.05)	0.652 (*p* < 0.05)
Dissolved oxygen	0.590 (*p* < 0.05)	0.594 (*p* < 0.05)	0.518 (*p* < 0.05)	0.614 (p < 0.05)

*Note*: Data were analyses based on 95% confident level

**TABLE 6:** Two-way Classification Analysis (TCA) results for sexual dimorphism of climbing perch, *Anabas tetsudineus* across all morphometric parameters.

Parameter	Factor	F	*p*	Sig	Notes/Post-hoc
Total length (cm)	Sex	12.46	0.008	**	Females > Males (12.04 ± 0.62 vs. 11.25 ± 0.66)
	Season	0.99	0.345	ns	–
	Habitat	15.90	0.002	**	Canal > Swamp > Paddy (all significant)
Body weight (g)	Sex	42.89	<0.001	***	Females > Males (38.67 ± 6.34 vs. 26.49 ± 2.89)
	Season	0.63	0.449	ns	–
	Habitat	12.31	0.007	**	Canal > Swamp > Paddy (all significant)
Condition factor	Sex	28.64	0.001	***	Females > Males (2.38 ± 0.25 vs. 1.74 ± 0.14)
	Season	0.45	0.521	ns	–
	Habitat	2.87	0.134	ns	–
Temperature (°C)	Season	89.23	<0.001	***	Dry > Rainy (31.2 ± 1.1 vs. 26.6 ± 0.8)
Dissolved oxygen (mg/L)	Season	15.68	0.001	***	Rainy > Dry
	Site type	6.43	0.009	**	Ditch > Pond > Paddy

*Note:*

*** *p* < 0.001,

** *p* < 0.01,

* *p* < 0.05, ns = not significant
